# Barriers to adherence to iron chelation therapy among adolescent with transfusion dependent thalassemia

**DOI:** 10.3389/fped.2022.951947

**Published:** 2022-10-06

**Authors:** Rafaa Mohamed, Amir Hamzah Abdul Rahman, Farin Masra, Zarina Abdul Latiff

**Affiliations:** ^1^Department of Pediatrics, Tengku Ampuan Rahimah Hospital, Klang, Malaysia; ^2^Department of Pediatrics, Hospital Tengku Ampuan Afzan, Kuantan, Pahang, Malaysia; ^3^Department of Pediatrics, National University of Malaysia, Bangi, Malaysia

**Keywords:** adherence, iron chelation, adolescents, thalassemia, transfusion-dependent

## Abstract

**Study background:**

Thalassemia is the commonest genetic blood disorder in Malaysia which requires life-long blood transfusions. From a total of 7,984 thalassemia patients in Malaysia, adolescent age group account for the highest number of patients (2,680 patients, 33.57%). In developed countries, the average rate of adherence to long-term treatment among children and adolescents is only 58%. Sub-optimal adherence to iron chelation therapy may impact the outcome and quality of life in these patients. Thus, assessing adherence level and identification of risk factors for non-adherence is essential in optimizing management.

**Objectives:**

To determine the association between mean serum ferritin level with self-reported level of adherence to iron chelation therapy in transfusion dependent thalassemia (TDT) adolescents in Hospital Tengku Ampuan Afzan (HTAA), Kuantan and Pusat Perubatan Universiti Kebangsaan Malaysia (PPUKM), Cheras; to determine the association between socio-demographic factors and patients’ knowledge on thalassemia and iron chelation therapy with the level of adherence.

**Materials and methods:**

This was a cross-sectional study conducted between 1st March 2019 and 31st March 2020. Data was collected through face-to-face interview by a single interviewer during the thalassemia clinic follow up, with content validated questionnaires. The questionnaires comprised four sections which included socio-demographic data, medication adherence questionnaire, knowledge of disease, and clinical characteristics of the participants.

**Results:**

A total of 70 participants were recruited. Results showed that only 51.4% of participants had good adherence to iron chelation therapy. There was a significant association between monthly household incomes of the family with the level of adherence to iron chelation (*p*-value 0.006). There was also an association between the mean serum ferritin levels with total Adherence Starts with Knowledge (ASK-12) score (*p*-value 0.001). However, there was no association between knowledge on thalassemia with the level of adherence.

**Conclusion:**

Adherence to iron chelation was generally unsatisfactory amongst adolescents with TDT as only 51.4% had good adherence. Low monthly household income of the family may affect adherence to iron chelation therapy in TDT patients. As adherence remains to be an issue amongst adolescent thalassemia patients, management should include regular and objective assessments to address this problem so as to optimize patient outcome.

## Introduction

Thalassemia is a severe public health problem in the Mediterranean, Middle East, Indian sub-continent, and South East Asia countries (1). Thalassemia is a heterogenous blood disorder characterized by partial or no production of alpha or beta globin chains. Although there is a wide spectrum of clinical phenotype, there are two main clinical forms: transfusion-dependent thalassemia (TDT) and non-transfusion dependent thalassemia (NTDT).

The main aim of transfusion is to maintain the mean hemoglobin level above 10°g/dL in order to suppress ineffective erythropoiesis. Allogeneic hematopoietic cell transplantation remains the only established definitive cure with thalassemia-free survival rates over more than 90% in selected young, low-risk patients (2). However, regular, lifelong blood transfusions and iron chelation therapy are necessary for those where bone marrow transplantation is not an option (3).

Data from the Malaysian Thalassemia Registry (May 2018) reported a total of 7,984 registered patients, of whom 5,420 were patients with transfusion-dependent beta thalassemia major and HbE beta thalassemia. The thalassemia intermedias account for 748 patients, HbH disease affects 1,458 individuals, and the remaining patients were with other subtypes. A recent study on the prevalence of growth and endocrine disorders in Malaysian children with TDT found that short stature was the most prevalent (40.2%), followed by pubertal disorders (14.6%), and hypoparathyroidism (12.3%) (4). The survival rate of thalassemia is strongly influenced by the iron burden, adherence to iron chelation therapy, and recurrent blood transfusions (5).

Close surveillance and assessment of iron overload are critical in establishing effective iron chelation regimes tailored to the specific needs of the individual patient. Measuring cardiac and liver iron with magnetic resonance image (MRI) using the T2* technique has been a recommended method for monitoring body iron overload in TDT. MRI facilitates the measurement of tissue iron in a non-invasive manner; however, MRI is not universally available. Serum ferritin reflects a total body iron, and serial measurements are useful for monitoring treatment. Although affected by several factors such as infection or inflammation, the long-term monitoring of serum ferritin remains the practical method for monitoring iron overload worldwide. Serum ferritin maintained below 1,000 μg/L significantly improves survival in addition to both heart and liver function (6).

Iron chelation has undergone dramatic improvements over the last decade. Adequate chelation of iron can only be achieved by regular use of subcutaneous iron chelation therapy with desferrioxamine methane-sulfonate (DFO) infusions prior to the advent of oral chelating agents (i.e., deferiprone-DFP and deferasirox-DFX) (5). Unfortunately, even with the administration of effective subcutaneous iron chelation therapy with DFO, more than 50% of patients died before they reach the age of 35, mostly due to poor adherence to subcutaneous chelation agents (5).

Adherence is generally defined as the extent to which a patient’s behavior (taking medications, implementing a diet, and/or embracing lifestyle changes) corresponding to a healthcare provider’s recommendations (7). Adherence is a complex phenomenon that embraces interrelated factors associated with individual patient’s health, condition, treatment, and environment, as well as psychological factors, all of which influence a patient’s adherence to a prescribed regimen. Poor adherence severely compromises the effectiveness of treatment (7). Intervention to improve adherence would have a significant positive return on investment through the primary prevention of risk factors and the secondary prevention of adverse health outcomes. The average rate of adherence to long-term treatment among children and adolescents in developed countries is only 58% (7). This is in relation to chronic diseases such as asthma, diabetes mellitus, and epilepsy. Several studies have suggested that adolescents have lower adherence relative to younger children. Therefore, it is necessary to determine the prevalence of adherence (8).

To date, there is no gold-standard method to measure medication-taking behavior. It is possible to classify methods of measuring adherence as a direct method and indirect method. Direct methods include directly observed therapy, drug concentration measurement in blood, and a biological marker measurement in the body. Indirect methods include patients’ self-report, pill counts, pharmacy fill data, electronic medication monitoring, and clinical response assessment of the patient. One of the significant indirect methods for measuring medication adherence and has been the most commonly used method in the clinical setting is the patient self-report or questionnaire (8).

Adherence Starts with Knowledge (ASK-12) survey was developed by GlaxoSmithKline in July 2008 to assess behavior and barriers related to treatment adherence. The ASK-12 survey is a validated patient-reported measure of barriers to medication adherence and adherence-related behavior. It is a generic instrument and applicable to patients irrespective of their medical conditions. The ASK-12 survey can be used for all age groups, including pediatric and adult patients. ASK-12 questionnaires were designed to address three domains or subscales, namely, inconvenience or forgetfulness (3 items), health beliefs (4 items), and behavior (5 items) (9). The total score of ASK-12 demonstrated adequate internal consistency reliability, with a Cronbach α of 0.75 (10).

Adherence to iron chelation therapy is important to prevent complications of iron overload. Nevertheless, in transfusion-dependent thalassemia patients, especially in adolescents, ensuring iron chelation therapy adherence is challenging. Adolescents have been defined by the World Health Organization (WHO) as individuals between the ages of 10 and 19. A stronger desire for autonomy, more time spent outside the home, and an increased desire to “fit in” with peers are typical developmental changes of adolescents. Therefore, they tend to resist or ignore the advice or guidance of health care personnel and parents but prefer to mimic their healthy peers by liberating themselves from medical constrictions. In addition to this, there is also marked psychosocial transition that has occurred during adolescence (11).

Other factors that are considered to complicate adherence among adolescents include family issues (size, income, parents’ education, the prevalence of children with chronic illness and parental involvement and supervision of their children’s treatment), and demographic and clinical variables for adolescents, such as age, illness severity, and disease knowledge (8, 12, 13). Side effects associated with iron chelation therapy, as well as the socially embarrassing nature of the condition, may also constitute additional barriers to adherence.

Adherence to iron chelation therapy is the key to survival in thalassemia patients (8). The most widely cited factors for non-adherence to DFO were the presence of side effects (mainly discomfort at the injection site), the practice of traditional and complementary medicine, and lack of family support. Not surprisingly, psychosocial support had a positive influence on adherence, whilst those from lower-income households negatively influenced adherence (3).

Since 2005, DFO and oral DFP have become freely available and accessible to patients, while DFX had been more accessible in 2012 (14). In 2006, the Malaysian government had set aside a total of RM25 million per year for the provision of free treatment to all thalassemia patients. According to the Clinical Practice Guideline (CPG) Management of Thalassemia 2009, the monthly medication cost for a 30 kg patient on DFO is estimated at RM763.20 compared to the estimated cost of RM 2553.88 for a patient on Deferasirox (15).

At present, all iron-chelating agents are readily available and accessible at government health facilities in Malaysia. However, DFP and DFX are limited and age-dependent. Oral chelating agents, which have been found to have fewer side effects and are associated with improved adherence, are not routinely available in government hospitals owing to cost implications. However, a recent cost-effective analysis comparing DFO and oral chelating agents has shown that over a longer period, the oral chelating agent can be cost-effective over DFO, as poor adherence in DFO frequently contributes to iron overload, associated complications, and higher costs in the future (3).

The purpose of this study was to determine barriers to adherence to iron chelation therapy in TDT adolescents. This study evaluated the association between socio-demographic factors, knowledge on thalassemia and mean serum ferritin level with the level of adherence to iron chelation therapy.

## Materials and methods

### Study design and population

This was a multi-center cross-sectional study involving Pusat Perubatan Universiti Kebangsaan Malaysia (PPUKM), Cheras, and Hospital Tengku Ampuan Afzan, Kuantan. This study was conducted between 1st March 2019 and 31st March 2020. Data were collected during thalassemia clinic sessions through face-to-face interviews conducted by an interviewer.

The inclusion criteria were all transfusion-dependent thalassemia patients (those with regular, 2 to 8 weekly blood transfusion interval), aged 10 to 19 years, and treated with iron chelation therapy. The exclusion criteria were patients with thalassemia-unrelated co-morbidities or cognitive or mental illness diagnosed by a psychiatrist.

### Study protocol

The questionnaire was prepared in two languages (English and Bahasa Melayu). It comprised four sections, which included socio-demographic data, medication adherence questionnaire, thalassemia knowledge, and clinical characteristics of participants.

Adherence was measured using Adherence Starts with Knowledge-12 (ASK-12) questionnaire. The ASK-12 questionnaire was translated into Bahasa Melayu and back-translated to English to ensure that the contents were intact. The questionnaire was then pre-tested for face validity on 10 patients. ASK-12 is a 12-item questionnaire that consisted of three domains related to medication adherence; inconvenience or forgetfulness, treatment beliefs, and behavior. Response to each item was scored on a five-point scale. The level of adherence was categorized as poor and good adherence, with a total (maximum) ASK-12 score of 60. Takemura et al. reported that the optimal cut-off value of the ASK-12 total score for discriminating non-adherent patients was 23 (9). In the case with a sub-scale score that indicates greater adherence difficulties, the cut-off scores are indicated by an inconvenience subscale of more than 12, a treatment belief of more than 16, and behavior of more than 20.

The knowledge questionnaire was adapted from the Disease Knowledge about Thalassemia Major (DKTM) questionnaire with permission (Al-Kloub et al.) (8). This questionnaire was administered to adolescents. Accompanying parents were not permitted to assist the patients responded to the questionnaire. The questionnaire comprised 20 true-false questions which measure patients’ knowledge and awareness of thalassemia. The score for each item was either 0 (incorrect/did not know) or 1 (correct). According to the study, good knowledge was reflected by more than 15 points, whereas poor knowledge was less than 15.

The section of clinical characteristics included thalassemia types, the age at diagnosis and mean serum ferritin level over the past one year. The targeted mean serum ferritin was less than 2,500°ug/L as per the Clinical Practice Guideline Management of TDT in Malaysia (15). Morbidities due to chronic iron overload were also recorded which included include short stature, hypothyroidism, delayed puberty, and diabetes mellitus. The results of MRI T2* (cardiac, liver) were included where available.

This study was approved by the Malaysian Research Ethics Committee (MREC) and the UKM Research Ethics Committee (UKMREC). Written informed consent was obtained from all parents and/or patients.

Calculation of sample size was performed using Power and Sample Size Program software version 3.1.6 [Dupont and Plummer, (16)]. For the objective that compared two proportion (level of adherence with knowledge), a sample size of 62 and 31 (adherence and non-adherence groups respectively), was required in order to detect the proportion difference of 21% with a power of 0.8 and alpha 0.05. Based on the results of a study by MI Al-Kloub et al. in 2014, the proportion of poor knowledge was estimated as 0.29. Thus, based on the calculation done on the objective of knowledge and adherence association, following a consideration of 20% non-response rate, the final sample size was 120 patients with a statistical power of more than 80%.

Statistical analysis was carried out by means of SPSS 26 (IBM SPSS Statistics). Results were presented in percentage for categorical data, mean (SD) for parametric numerical data, and median (IQR) for non-parametric continuous data. Chi-square test analysis was conducted to identify significant factors affecting the adherence level to iron chelation therapy, and thalassemia knowledge. The Mann-Whitney *U* Test assessed non-parametric numerical variables. Spearman’s Rho Correlation Test identified the correlation between mean serum ferritin level and ASK-12 total score and subscales. The normality test for the parameters was analyzed with the Kolmogorov-Smirnov Test and Shapiro-Wilk Test. Statistical significance was defined at a *p*-value of less than 0.05.

## Results

A total of 70 patients were enrolled during the study period; 39 patients from Hospital Tengku Ampuan Afzan (HTAA), and 41 patients from Pusat Perubatan Universiti Kebangsaan Malaysia (PPUKM). Table 1 summarizes the socio-demographic data and clinical characteristics of patients. The mean age was 14.23 (SD ± 2.93) years, and respondents were predominantly males (52.9%) followed by females (47.1%). The majority of patients were Malays (85.7%). All patients received formal education. Approximately half of the parents received secondary-school education level (mother, 52.9%; father, 54.3%). Forty percent of families earned less than RM 3000 per month (Table 1).

**TABLE 1:** Socio-demographic and clinical characteristics of 70 thalassemia patients.

Characteristics	n (%)
**Age (mean 14.23, SD ± 2.93, years)**	
**Gender**	
Male	37 (52.9)
Female	33 (47.1)
**Ethnic**	
Malay	60 (85.7)
Chinese	9 (12.9)
Others (Bidayuh)	1 (1.4)
**Patient’s education level**	
Primary school	25 (35.7)
Secondary school	45 (64.3)
**Maternal highest education level**	
None	1 (1.4)
Primary school	6 (8.6)
Secondary school	37 (52.9)
Tertiary*	26 (37.1)
**Paternal highest education level**	
None	1 (1.4)
Primary school	4 (5.7)
Secondary school	38 (54.3)
Tertiary*	27 (38.6)
**Monthly household income**	
Less than RM 3000	28 (40)
RM 3000 till RM 5000	20 (28.6)
More than RM 5000	22 (31.4)
**Diagnosis**	
Homozygous beta thalassemia	21 (30)
Beta-thalassemia intermedia	6 (8.6)
HbE-beta thalassemia	37 (52.9)
HbH disease	4 (5.7)
Alpha thalassemia (Hb adana constant spring)	2 (2.9)
**Age of diagnosis (mean 3.04, ± SD 2.33, years)**	
**Serum ferritin level, μ g/L (mean 2229.89, ± SD 1491.56)**	
**Iron chelation**	
Monotherapy	60 (85.7)
Combination therapy	10 (14.3)
DFO and DFP	8 (11.4)
DFO and DFX	1 (1.4)
DFP and DFX	1 (1.4)
**Co-morbidities related to thalassemia**	
Diabetes mellitus	2 (2.9)
Hypothyroidism	4 (5.7)
Short stature	29 (41.4)
Delayed puberty	8 (11.4)

*Tertiary: college/university education. RM, ringgit Malaysia; DFO, Desferrioxamine; DFX, Deferasirox; DFP, Deferiprone; SD, standard deviation.

HbE-beta thalassemia was diagnosed in 52.9% of patients. The mean age of diagnosis was 3°years old. The majority (85.7%) of patients was on monotherapy; 50% were on deferasirox. The remaining 10 patients (14.3%) were on combination therapy of which 80% were on desferrioxamine and deferiprone. The mean serum ferritin level was 2229.89 (SD ± 1491.56). A total of 68.6% of patients had MRI T2* performed in the past one to 2°years. Among these, 62.9% of them had no detectable iron deposition in the myocardium, whereas 18.6% had no iron deposition in the liver. The remaining patients (81.4%) had a variable level of iron deposition in the liver, ranging from mild to severe.

Of the 70 patients, 51.4% had good adherence to iron chelation therapy. The score ranged from 21 to 50, with a mean score of 31.31 (SD ± 4.75). Only one patient (1.4%) had a score of more than 16 for the treatment belief sub-scales and more than 20 for the behavior sub-scales. There was statistical correlation between age of patients and total ASK-12 score, with an *r*-value of 0.28 and *p*-value of 0.017. There was also statistical correlation between mean serum ferritin level and total ASK-12 score, with an *r*-value of 0.40 and *p*-value of 0.001. Whereas for the sub-scales analysis of ASK-12 (Table 2), there was a correlation between inconvenience/forgetfulness and behavior subscales with the mean serum ferritin level (*p*-values of 0.021 and 0.007, respectively).

**TABLE 2:** Correlation between ASK-12 and its subscales with mean serum ferritin level.

Scores	Mean serum ferritin	
	*r*	*P-value*
Inconvenience/forgetfulness	0.28	0.021*
Treatment beliefs	0.21	0.080
Behavior	0.32	0.007*
ASK-12 total score	0.40	0.001*

*Positive correlation with *p*-value.

The average score for thalassemia knowledge as measured by the DKTM scale was 16.77 (± SD 2.37, range 8 to 20). Results revealed that 80% of participants scored 15 and above, indicating a high level of knowledge on thalassemia. The majority (82.9%) of patients were unable to respond correctly to Question 3 (“Each patient with thalassemia needs blood transfusion treatment”). Almost all of the patients (98.6%) were aware that accumulation of iron will result in cardiomyopathy.

The association of adherence to socio-demographic factors, clinical characteristics, and knowledge of thalassemia were summarized in Table 3. There was a statistically significant association between adherence and low monthly household income (*p*-value of 0.006).

**TABLE 3:** Association of socio-demographic and clinical factors with level of adherence to iron chelation therapy amongst 70 thalassemia patients.

Factors		ASK-12 total score		*P-value*
		Good	Poor	
		n (%)	n (%)	
Gender	Male	17 (45.9)	20 (54.1)	0.33
	Female	19 (57.6)	14 (42.4)	
Ethnic	Malay	33 (55)	27 (45)	0.08
	Chinese	2 (22.2)	7 (77.8.9)	
Patient’s education level	Primary school	16 (64)	9 (36)	0.11
	Secondary school	20 (44.4)	25 (55.6)	
Maternal highest education level	None	0 (0)	1 (100)	0.53
	Primary school	4 (66.7)	2 (33.3)	
	Secondary school	18 (48.6)	19 (51.4)	
	Tertiary*	14 (53.8)	12 (46.2)	
Paternal highest education level	None	0 (0)	1 (100)	0.68
	Primary school	2 (50)	2 (50)	
	Secondary school	20 (52.6)	18 (47.4)	
	Tertiary*	14 (51.9)	13 (48.1)	
Monthly household income	Less than RM3000	10 (35.7)	18 (64.3)	0.006*
	RM3000 till RM5000	16 (80)	4 (20)	
	More than RM5000	10 (45.5)	12 (54.5)	
Iron chelation	Monotherapy	32 (53.3)	28 (46.7)	0.43
	Combination therapy	4 (40)	6 (60)	
Level of knowledge on thalassemia	Poor (< 15)	7 (50)	7 (50)	0.90
	Good (≥ 15)	29 (51.8)	27 (48.2)	
Co-morbidities related to thalassemia	None	14 (39.9)	20 (58.8)	0.09
	Yes	22 (61.1)	14 (41.2)	

*Tertiary: college/university education.

## Discussion

Adherence to chelation therapy is the key to survival and longevity in patients with thalassemia (8). Huang et al. (17) found that good adherence to iron chelation increased life expectancy up to 46 years of age (17). Adolescents are more likely to be non-adherent compared to children under 10 years of age (8). This may be contributed by unique changes that occur during adolescence. In adolescence, their sense of control over their lives, cognitive limitations of risk evaluation, and relative lack of experience with long-term consequences may lead to the belief that they do not need to follow treatment plans.(8) Dependency on doctors and caregivers, created by the need for medical treatment, appears to threaten the adolescents’ desire for autonomy, which may subsequently lead to rejection of treatment recommendations (8).

The results of our study revealed that adherence to iron chelation therapy amongst adolescents with TDT was sub-optimal, with only 51.4% having good adherence to iron chelation. Lee et al. (3) reported that 81.2% had good adherence. However, assessment of adherence was based on the administration frequency of DFO over a period of 1°month, and no other research instruments were employed to measure prior adherence level.

Our study found that a low monthly household income of less than RM 3000 was associated with poor medication adherence. Although the Malaysian government has subsidized oral chelators since 2006, the provision of newer oral chelators (mainly deferasirox) relies on the overall budget allocated to a particular hospital. In our study, all patients who received deferasirox were fully funded by the hospital. However, the provision of adequate equipment such as infusion pump, needles, and syringes, may still incur additional costs for the family. Furthermore, additional costs, such as transportation expenses, may also pose a significant burden, especially for low- and middle-income families. It is estimated that patients spend approximately RM 150 to RM 200 per month (RM 50 for transports, and the remainder for medical equipment) on these additional expenses.

Our results showed that eighty percent of the patients had a clear understanding of thalassemia. Lau et al. (18) reported that only 10.8% of patients with thalassemia in their study cohort had a good comprehension of their disease (18). Although their questionnaire was different, it nonetheless showed a positive pattern as patients became more aware of their illness and the implications of the disease. They were aware of the disease, treatment, and complications that could result from progression of the disease. Our study found no association between the adherence level and knowledge on thalassemia, as reported in a prior study by Al-Kloub et al. (8). Awareness of the disease and its treatment is essential for patient adherence; having said that, knowledge or awareness alone is not adequate to promote healthy and positive behavior toward adherence. Thus, healthcare providers should devote additional efforts to empower their patients with knowledge on thalassemia and increase awareness of the importance of adherence to iron chelation.

The long-term outcome of patients with transfusion-dependent thalassemia depends on frequent and adequate blood transfusions and the prevention of excessive iron overload (3). As MRI T2* of the myocardium and liver is not readily available, the measurement of serum ferritin levels, albeit its limitations, is still widely used as an indicator of total body iron load. In our study, there was an almost significant statistical correlation (*p* value of 0.054) between the level of adherence and mean serum ferritin level (Table 4). This is in contrast to a study by Lee et al. (3) which showed no correlation between the level of adherence and mean serum ferritin level (*p*-value of 0.186). The mean serum ferritin level of children with thalassemia in the study by Lee et al. (3), was much higher than the recommended range following chelation therapy. The median age of their patients was 10 years old, whilst the median age at diagnosis was 1°year old. This infers that these patients have had an average of 9°years of regular blood transfusions. However, as DFO was only fully subsidized 2°years prior to their study for all patients, it is not surprising then that the mean serum ferritin level was reportedly higher.

**TABLE 4:** Association of level of adherence toward iron chelation with serum ferritin level.

Level of adherence	Serum ferritin level (μ g/L)			
	All patients n (%)	Less than 2,500 n (%)	More than 2,500 n (%)	*P-value*
*Good	36 (51.4)	27 (60)	9 (36)	0.054
*Poor	34 (48.6)	18 (40)	16 (64)	

*ASK-12 total score: Good adherence < 23, poor adherence ≥ 23.

Combination therapy for iron overload treatment was introduced in patients with iron overload who are sub-optimally chelated with monotherapy. Treatment with two chelators may improve organ-specific iron removal, reduce toxicity, and enhance iron removal if there is an additive or synergistic effect (19). Our study found no association between the levels of adherence to iron chelation treatment received by patients. There was, however, a significant association between mean serum ferritin levels and combination therapy. Higher serum ferritin level in patients on combination therapy (desferrioxamine and deferiprone) may have resulted from poor adherence as lower serum ferritin is expected in these patients. Adverse effects of iron chelation such as irritation or swelling at the infusion site and gastrointestinal symptoms such as nausea, vomiting, diarrhea and abdominal pain may have contributed to poor adherence.

There were several limitations to this study. This study was constrained by a small sample size, which restricts sound statistical conclusions on the determination of adherence-related risk factors. The sample size was relatively smaller as the number of patients, from both recruitment centers, within the age range of 10 to 19 years was limited. This can be overcome by recruiting more participants from other health centers. In addition to this, the study was conducted through direct interviews with the patients themselves, which may have potentially have led to bias in responses.

Serum ferritin level has been employed as an indicator of chronic iron overload as MRI T2* is not readily available. The use of serum ferritin level is, nevertheless, not an ideal indicator due to various factors such as different medication absorption and blood transfusion rates, which may affect total body iron level (e.g., liver diseases and ascorbic acid deficiency).

## Conclusion

Adherence to iron chelation is generally unsatisfactory amongst adolescents with transfusion-dependent thalassemia; only 51.4% of patients had good adherence. Low monthly household income was associated with poor adherence to iron chelation. There was no correlation between knowledge on thalassemia and adherence levels.

Our results have significant implications for the clinical management of adolescents and young adults with thalassemia. Clinicians need to assess, monitor routinely, and encourage adherence behavior. Clinicians should integrate multiple objective methods when assessing adherence. In cases where adherence is a challenge, a systemic approach should be adopted with careful consideration of family-specific factors and psychological issues. This includes regular assessments using a validated transition readiness questionnaire (that includes issues related to adherence) in addition to incorporating a multidisciplinary clinic with the involvement of certified pharmacists who will facilitate counseling related to adherence and medications. A more concerted effort should also be made to assist adolescents with lower socioeconomic status as they are at a higher risk of non-adherence. This includes referral to social welfare or substitution of DFO with oral chelating agents.

## Data availability statement

The raw data supporting the conclusions of this article will be made available by the authors, without undue reservation.

## Ethics statement

The studies involving human participants were reviewed and approved by Universiti Kebangsaan Malaysia Research Ethics Committee. Written informed consent to participate in this study was provided by the participants or their legal guardian/next of kin. Our study was approved by the Medical Research and Ethics Committee of Malaysia (Research ID NMRR-18-2690-43731) and Research Ethics Committee Universiti Kebangsaan Malaysia (Project code JEP-2018-650).

## Author contributions

ZA: conceptualization, supervision, manuscript writing, review, and editing. FM: conceptualization, manuscript writing, review, and proofreading. AA: conceptualization, supervision, and manuscript writing. RM: conceptualization, conduct of research, and manuscript writing. All authors contributed to the article and approved the submitted version.
